# Are we too far from being client centered?

**DOI:** 10.1371/journal.pone.0205681

**Published:** 2018-10-15

**Authors:** Belay Erchafo, Tesfamichael Alaro, Gebeyehu Tsega, Ayinengida Adamu, Kiddus Yitbarek, Yibeltal Siraneh, Meaza Hailu, Mirkuzie Woldie

**Affiliations:** 1 Department of Public Health, College of Medicine and Health Sciences, Wachemo University, Hosaena, Ethiopia; 2 Department of Health Policy and Management, Faculty of Public Health, Jimma University, Jimma, Ethiopia; 3 Department of Health Service Management, College of Public Health, Bahirdar University, Bahirdar, Ethiopia; 4 Health Service Quality Division, Oromia Regional State Health Bureau, Addis Ababa, Ethiopia; 5 Maternal and Child Health Directorate, Federal Ministry of Health, Addis Ababa, Ethiopia; Aga Khan University, KENYA

## Abstract

**Background:**

Quality of service provision in health facilities is fundamental to ensure effective care. However, women’s actual experience of care is often neglected.

**Objective:**

To assess perceived quality of institutional delivery services and associated factors among women who delivered in public health facilities of Southwest Ethiopia.

**Method:**

Community based cross-sectional study was conducted in three districts of Jimma zone, Southwestern Ethiopia, from February 29 to March 20, 2016. A total of 423 mothers who delivered in public health facilities during the last 12 months were selected to participate in the study. Study participants were identified using simple random sampling procedure. Principal component analysis was used to generate scores for three sub-dimensions of perceived quality. Multiple linear regression analysis was performed to identify predictors of these sub-dimensions.

**Results:**

Perceived quality of institutional delivery services was measured with three dimensions: perceived interpersonal interaction, health care delivery and health facility/structure. We found that perceived quality of interpersonal interaction was negatively affected by educational level (read and write) (β: -0.331, 95% CI: -0.523, -0.140), urban residence (β: -0.485, 95% CI: -0.696, -0.275), antenatal care (less than three visits) (β: -0.238, 95% CI: -0.419,-0.056) and delivery service attended by male provider (β: -1.286, 95% CI: -1.463,-1.109). Perceived quality of health care delivery was negatively associated with still birth (β: -0.642, 95% CI: -1.092,-0.193) and delivery services attended by male provider (β: -0.689, 95% CI: -0.907,-0.472). Urban residence (β: -0.260, 95% CI: -0.515,-0.005), and antenatal care (less than three visits) (β: -0.394, 95% CI: -0.628,-0.161) were negatively associated with perceived quality of health facility/structure.

**Conclusion:**

Overall, the perceived quality of institutional delivery services was low. We recommend that health managers and health care providers jointly work to transform birth care at the health facilities to deliver person-centered care. Addressing the preferences of clients is as important as taking care of structural concerns pinpointed in this study.

## Introduction

Developing regions account for approximately 99% (302,000) of the global maternal deaths which is roughly 20 times higher than that of developed regions, whereas sub-Saharan Africa (SSA) alone accounting for roughly 66% (201,000) [1,2]. In Ethiopia, according to recent World Health Organization (WHO) estimate proportion of mothers who were dying to give 100,000 live births has declined to 353 in 2015 from 420 in 2013. Achieving the SDG target of a global Maternal Mortality Ratio (MMR) below 70 will require reducing global MMR by an average of 7.5% each year between 2016 and 2030 [3]. This requires more than three times the 2.3% annual rate of reduction observed globally between 1990 and 2015 [4].

It is recognized that skilled delivery assisted by professionals results in significant reduction in maternal mortality [5]. However, the provision of appropriate maternal health care remains one of the main challenges in developing countries [6]. In Ethiopia, only sixteen percent of births were assisted by a skilled provider whereas fifty-one and twenty-seven percent of births were assisted by a relative, or some other person and traditional birth attendants, respectively [7]. This has only marginally improved in the latest Ethiopian Demography and Health Survey (EDHS) report [8]

Women call for high quality client-oriented care services that address their individual needs throughout pregnancy in order to guarantee optimal health for them and their babies [9]. Perception of low quality has been reported as a major factor in non-utilization or bypassing of health services [10–12]. Often the birth place of a child decided by the mother and her intimates referring their evaluation of service from their experience [13].

Health care quality can be defined as methods and procedures coherent with the existing professional knowledge applied to individuals and the community so as to increase the possibility of desired health outcome [14]. It can be considered from the provider or user’s perspective, and is differentiated into observed and perceived quality [15,16]. Users, in reality, play a central role in defining and assessing quality of care because they choose whether or not to go for care based on their opinions, informed by their previous experiences with the health system and those of people they know [17]. It becomes critical to define and measure client perceptions of health care quality and to understand more fully what drives those perceptions [18].

Perceived quality of institutional delivery services generally remains poor in most public health facilities in Ethiopia [19]. Use of a facility has been highly influenced by perceived quality, which was mainly determined by the competence of health workers and the relationship with the health workers, as well as the facility amenities [20]. While the quality of the provision of care in facilities is fundamental to ensure effective care, women’s actual experience of care is a significant, but often neglected aspect of quality of care that contributes to maternity outcomes [21]. The current priority agenda for maternal health service in general and delivery service in particular is client centeredness [22].

Audit of health care quality from the perspective of clients and patients is becoming more and more important. The call for person-centered care to ensure uptake and continuity of care seeking along the continuum of maternal and child care necessitates looking at quality from the client’s perspective. While clients may not be able to evaluate whether a specific technical procedure is appropriate, they can assess quality according to the availability of medical equipment and the behavior of the health care provider.

Despite the fact that institutional delivery care quality is essential for further improvement of maternal and child health; little is known about the current perceived quality of the institutional delivery services and factors influencing the perceived quality of care in Ethiopia. Therefore, the purpose of this study is to assess the perceived quality of institutional delivery services provision and identify factors influencing perceived quality in public health institutions of Southwest Ethiopia.

## Methods

### Study setting and participants

This study was conducted among women who gave birth at public health facilities of three of the 18 districts in Jimma zone, Oromia Regional State, Southwest Ethiopia. In the zone there are 5 primary hospitals, 115 health centers and 520 health posts. Sample size was determined using single population proportion formula where the proportion of high perceived quality with institutional delivery was assumed to be 50%, with 5% margin of error and confidence interval of 95%. Participants were taken from all mothers who gave birth within the last 12 months in public health facilities in the three districts. Ten kebeles (lowest administrative unit) were selected randomly from three districts of Jimma zone: Gomma woreda (4), Kersa woreda(3) and Seka Chekoresa woreda (3). Complete list of mothers who gave birth during the last 12 months was obtained from health extension registration book at the health post located in each kebele. The total sample of 423 was distributed to the ten kebeles proportionally based on the number of households.

### Measurement and data collection

Questionnaire for interview was adapted from studies conducted in other developing countries and was contextualized to the study setting [23–25]. Perceived quality of institutional delivery services was measured using five point Likert scales containing 20 items. Principal component analysis resulted in retention of 18 of these items organized into three sub-dimensions. The sub-dimensions represented interpersonal interaction (8 items), health care delivery (4 items) and health facility/structure (6 items). The total variance explained by these factors was 43.95%. Adding more factors to these sub-dimensions would increase the total variance explained but also decreases its practicality. So, rather than missing its practicality we preferred to act on identified the three sub-dimensions [23,25,26]. We have confirmed the internal consistency of The Cronbach’s alpha confirmed internal consistency of the three sub-scales, ranged from 0.68 to 0.77.

The data collection tool also captured background variables such as age, religion, educational status, marital status, residence, occupation and wealth index. Data were also collected on parity, number of antenatal care (ANC) visits, route of delivery of the last child, any complication during delivery of the last child, type of institution used, sex of the care provider who attended the delivery, duration of labour, outcome of birth and history of abortion. The data collection was conducted after ethical clearance and letter of support was obtained from the institutional review board (IRB) of Jimma University.

### Data processing and analysis

Data were entered into EpiData version 3.1 and exported into SPSS version 16 for analysis. All variables with p-value less than 0.25 in bi-variate analysis were considered as candidates for multiple linear regressions analysis. Multivariate linear regression analysis was done through enter method to identify the most significant predictors. Significant independent predictors were declared at 95% confidence interval and P-value of less than 0.05 and unstandardized β was used for interpretation.

Percentage mean score for perceived quality of institutional delivery service was calculate using the formula below [27,28]:

$$Percentage\;mean\;score=\frac{Actual\;score-Potential\;minimum\;score}{Potential\;maximum-Potential\;minimum}*100$$

In the descriptive presentation of findings from the Likert scales, we have described responses of agree and strongly agree as positively perceived and the other three response categories (neutral, disagree and strongly disagree) as negatively perceived.

## Results

### Socio-demographic characteristics

Four hundred eleven respondents were interviewed. The mean age of respondents was 28 ± 4.6 years. Three hundred eighteen (77.4%) of the mothers were from rural areas. Three hundred (73.0%) of mothers reported exposure to radio or television messages. It took less than an hour for 78.1% of the mothers to reach to the public health institutions they gave birth at (Table 1).

**Table 1:** Socio-demographic characteristics of women who gave birth in public health facilities, Southwest Ethiopia, 2016.

Variables	Categories	Frequency	Percent
Residence	Rural	318	77.4
	Urban	93	22.6
Religion	Muslim	340	82.7
	Orthodox	60	14.6
	Protestant	11	2.7
Ethnicity	Oromo	336	81.8
	Amhara	28	6.8
	Kefa	18	4.4
	Dawuro	17	4.1
	Others*	12	2.9
Age	<20	5	1.2
	20–34	362	88.1
	35–49	44	10.7
Occupation	House wife	332	80.8
	Merchant	32	7.8
	Farmer	31	7.5
	Daily laborer	10	2.4
	Government employee	6	1.5
Educational status	Unable to read and write	181	44.0
	Only read and write	109	26.5
	Primary education	92	22.4
	Secondary education	24	5.8
	College and above	5	1.2
Marital status	Married	404	98.3
	Others**	7	1.7
Wealth index	Lowest	82	19.95
	Second	82	19.95
	Middle	83	20.20
	Fourth	82	19.95
	Highest	82	19.95

* Others = Gurage, Yem.

** Others = single, divorced and widowed.

### Perceived quality of institutional delivery service

The percentage mean score of perceived quality of care as measured by the interpersonal interaction sub-scale was 63%. More than half of the participants (51.1%) positively rated the adequacy of the time allocated by health care provider during labor and delivery. Adequacy of information provided to mothers during labor and delivery were perceived positively by 61% of the study participants. Two hundred eighty-two (69%) of the study participants reported on positive experience about provision of respectful service during labor and delivery.

The percentage mean score of perceived quality of care as measured by the health care delivery sub-dimension was 70%. We found that 349 (85%) of the mothers perceived their privacy during labor and delivery positively. Ease of drug availability was also perceived positively by 251 (61%) of the women interviewed.

The percentage mean score of perceived quality of delivery service as measured by the health facility/structure sub-scale was 58%. Two hundred forty-two (59%) of the mothers had positive perception about the adequacy of the delivery room. Half of the study participants positively perceived the cleanliness of the facility they used. However, only 28% of the interviewed mothers had positive perception about the adequacy of water supply in the public health institutions they used during their last delivery (Table 2).

**Table 2:** Perceived quality of institutional delivery service in three dimensions among women who gave birth in public health facilities, Southwest Ethiopia, 2016.

Items	Positive (%)
**Interpersonal interaction dimensions**	
Well examination of health staffs	281 (68.37)
Adequacy of information during delivery	249 (60.58)
Capability of health staffs to finding out what is wrong with the mothers	299 (72.75)
Compassion for mothers	263 (63.99)
Honesty of health staffs	289 (70.32)
Openness of health staffs with mothers	298 (72.51)
Respectfulness of health staffs towards the mothers	284 (69.10)
Adequacy of time the health staffs devoted to mothers	210 (51.09)
**Health facility/structure dimension**	
Health staffs suitability to treat women health problems	335 (81.51)
Adequacy of delivery room	242 (58.88)
Adequacy of water for women in the facility	123 (29.93)
Cleanliness of the health facility	221 (53.77)
Equipment suitability	216 (52.55)
Health staff adequacy	310 (75.43)
**Health care delivery dimension**	
Privacy during delivery	349 (84.91)
Needed drugs prescription	332 (80.78)
Good drugs supply	329 (80.05)
Easy availability of drugs	251 (61.07)

### Determinates of perceived quality of delivery services

#### Perceived quality of interpersonal interaction

Interpersonal interaction score was significantly lower for mothers who were able to read and write relative to those who were unable to read and write (β: -0.331, 95% CI: -0.523, -0.140). Similarly, urban residents (β: -0.485, 95% CI: -0.696, -0.275), those who attended less than three ANC visits (β: -0.238, 95% CI: -0.419, -0.056) and those who had their deliveries attended by a male provider (β: -1.286,95% CI: -1.463, -1.102) had significantly lower interpersonal interaction scores. However, interpersonal interaction score was significantly higher for mothers who were in the second wealth quintile as compared to those in the middle (β: 0.278, 95% CI: 0.04–0.516) (Table 3).

**Table 3:** Predictors of perceived quality of institutional delivery service among women who gave birth in public health facilities, Southwest Ethiopia, 2016.

Categories	Unstandardized β (95% CI) of interpersonal interaction dimension	Unstandardized β (95% CI) of health care delivery dimension	Unstandardized β (95% CI) health facility/ structure dimension
**Residence**			
Urban	-.485 (-.696, -.275)*		-.260 (-.515, -.005)*
Rural^&^			
**Listen to radio or watch television**			
No	.073 (-.105, .252)	.103 (-.108, .313)	.013 (-.213, .240)
Yes^&^			
**Parity**			
1	.025 (-.199, .248)	.017 (-.250, .284)	-.021 (-.302, .259)
1–4	.001 (-.183, .181)	-.024 (-.243, .194)	.023 (-.209, .256)
6 or more	-.095 (-.377, .187)	-.082 (-.419, .255)	-.069 (-.431, .293)
2–3^&^			
**Abortion experience**			
Yes	-.094 (-.360, .172)	-.166 (-.145, .477)	.170 (-.167, .507)
No^&^			
**Frequency of ANC visits**			
Less than 3 visits	-.238 (-.419, -.056)*	.197 (-.023, .416)	-.394 (-.628, -.161)*
3 or more visits^&^			
**Mode of delivery**			
Normal vaginal delivery^&^			
Assisted delivery	-.204 (-.519, .112)	-.072 (-.455, .310)	-.009 (-.406, .387)
Cesarean section	.215 (-.119, .549)	.207 (-.194, .608)	.019 (-.384, .423)
**Sex of health care provider**			
Male	-1.286 (-1.463, -1.109)*	-.689 (-.907, -.472)*	.091 (-.137, .320)
Female^&^			
**Duration of labor**			
Less than 12 hours^&^			
12–24 hours	.180 (-.046, .407)	-.176 (-.455, .102)	
Greater than 24 hours	-0.052 (-.484, .381)	-.187 (-.713, .340)	
**Fetal outcome**			
Still birth	-0.299 (-.665, .067)	-.642 (-1.092, -.193)*	-.063 (-.536, .410)
Live birth^&^			
**Wealth index**			
Middle^&^			
Lowest	0.077 (-.163, .316)		
Second	.278 (.040, .516)*		
Fourth	.106 (-.134, .347)		
Highest	-.118 (-.360, .125)		
**Time to the nearest facility**			
Less than 1 hour^&^			
1–2 hours	-.064 (-.263, .135)		
More than 2 hours	.0057 (-.499, .613)		
**Educational level**			
Unable to read and write^&^			
Read and write	-.331 (-.523, -.140)*		
Primary education	-.003 (-.216, .210)		
Secondary education	.047 (-.300, .395)		
College and above	.189 (-.526, .904)		

*Statistically significant at p-value < 0.05, 95% CI.

β- Positive values indicate higher quality score relative to the referent variable category level, while negative values indicate lower quality score compared to the referent category.

^&^reference category

#### Perceived quality of health care delivery

In the multivariable model, none of the socio-demographic factors were independent predictors of perceived quality of care as measured by the health care sub-scale. Health care delivery score was significantly lower for mothers who had their delivery attended by a male health care provider (β: -0.689, 95% CI: -0.907, -0.472). Expectedly, women who had still birth as compared to those who had a live birth also had significantly lower score (β: -0.642, 95% CI: -1.092, -0.193) (Table 3).

#### Perceived quality of health facility structure

Health facility/structure score was significantly lower for mothers who were from urban areas (β: -0.260, 95% CI: -0.515,-0.005) and those who had less than 3 ANC visits (β: -0.394, 95% CI: -0.628,-0.161) (Table 3).

## Discussion

We found that the percentage mean scores of perceived quality of institutional delivery service ranged from 58% to 70% based on which sub-scale was used to measure it. While the least perceived level of quality was reported for the health facility/structure sub-scale, the highest percentage was reported for perceived quality of health care delivery. Higher percentage was reported for this dimension because perception about health care delivery could be influenced by the fact that maternal health services including delivery care are exempted from payment in Ethiopia. However, the structure/facility component would get a much lower score since public health facilities are poorly equipped with the necessary supplies and equipment.

Respondents negatively perceived the interaction they had with the health care provider while receiving care. This was also the case in earlier studies where disrespectful behavior of health care providers negatively affected client satisfaction and interaction was reported [24,29]. Health care planners and providers may strive to improve quality of delivery service focusing on technical aspects [30]. However, the perception of mothers about the quality of delivery care they received may not always much with such improvements. Clients would definitely give more weight to a respectful and compassionate approach irrespective of the technical undertakings during delivery. Improving interpersonal interaction is, therefore, a promising way to improve perceived level of health care quality by clients [29].

Another area of concern noted in our findings relate to the duration of time allotted and the adequacy of information provided during delivery care. We found that only about half of the women favorably rated the adequacy of time they spent with the provider and less than two third agreed/strongly agreed that the information provided was adequate. Similar figure was reported from an earlier study from a referral hospital in northern Ethiopia [31]. However, a much higher proportion of women (92%) were content with the information received from the health care provider in a study from Iran [32]. Our findings are worrisome since failure to capture this opportunity to adequately inform on issues related to child care and family planning would have a bearing on the future of the women and her newborn. This if of course in addition to the effect of inadequate information and time on the delivery care itself.

In this study 31% of the participants did not feel that the service they received during labor and delivery was respectful. This is of concern since earlier studies have shown that disrespect and abuse during delivery is associated significantly with lower quality scores [33]. A study reported that women who had any disrespectful and abusive treatment during childbirth were less likely to rate the quality of care for delivery as excellent or very good [34].

Our respondents rated the quality of health facility/structure as low. Cleanliness of the health facility, adequacy of water supply and rooms for labor and delivery were considered as unsatisfactory by the clients. Similar concerns about quality of health facility structure including bed, toilet and space of public facilities were reported in studies from India and Nepal [24,29,35]. This is a concern since it is well documented that the physical environment of health facilities can impact on client perceived quality of care [35].

We found that educational status of the women in our study was significant predictor of perceived quality of institutional delivery services. Mothers who were able to read and write reported lower quality of interpersonal interaction than those who were unable to read and write. Comparable finding was reported in an earlier study where women who had completed secondary school rated the quality of delivery care lower than women with lower level of education [29]. This may seem obviously related to the fact that more educated and informed women would have higher expectations when visiting a health care provider. This may also hold true for the fact that women from urban areas had lower perceived quality of delivery care as compared to their rural counter parts both in our study and in a study from Uganda [36]. However, the study from Uganda reported that quality of care was significantly higher for post-secondary women relative to those with no education [36]. The more educated clients in this study may have been adequately versed about the processes during care which may help them to make more rational/pragmatic judgement about the quality of the care they received.

As in earlier studies [37,38], we have noted that perceived quality of institutional delivery services were higher for mothers whose delivery was attended by female health care provider. Although a part of this preference may be explained by religious reasons, it is also natural that a women would prefer a female health care provider in this particular occasion at least for two reasons. First, she may simply be more comfortable to have private parts exposed to a person with the same sex. Secondly, a female provider may be considered more compassionate and understanding to the laboring mother for she could be in the same circumstance at some point in her life.

Our study also showed that more ANC visits improved perception about the quality of delivery service. We noted that mothers who had 3 or more visits had higher scores of perceived quality of institutional delivery services as compared who had less. This finding is supported by an earlier report from Tanzania [33]. Hence, earlier and more frequent interactions with health care providers could help the women to establish rapport with the providers and feel more comfortable in their hands.

The findings reported in this study must be interpreted with the following limitations in mind. First, recall bias, is a possibility since mothers were asked about what they experienced in the health facility within the previous one year. Moreover, mothers who may have given birth in a health facility but not registered on the health extension worker’s registration book were not included in the study.

## Conclusion

Findings we have reported inhere imply that quality of institutional delivery care (perceived quality of interpersonal interaction and health facility structure) in public health facilities of the study area was not appealing to the clients. However, the perception of the women about the health care delivery process was reassuring. Building on the positive perception about the process of health care delivery by coaching and mentoring health care providers to improve interpersonal interaction during care will be helpful. We recommend that health managers and health care providers jointly work to transform birth care at the health facilities to deliver person-centered care. Addressing the preferences of clients is as important as taking care of structural concerns pinpointed in this study.

## Supporting information

S1 Table Characteristics of the three dimension principal component analysis (factorial loading) of selected items and Cronbach’s alpha values on each dimension in public health institutions of three districts of Jimma zone, southwest Ethiopia, 2016. (DOCX)

S2 Table Participants’ response on perceived quality of institutional delivery services on interpersonal interaction dimension in public health institutions of three districts of Jimma zone, southwest Ethiopia, 2016. (DOCX)

S3 Table Participants’ response on perceived quality of institutional delivery services on health facility/structure dimensions in public health institutions of three districts of Jimma zone, southwest Ethiopia, 2016. (DOCX)

S4 Table Participants’ response on perceived quality of institutional delivery services on health care delivery dimension in public health institutions of three districts of Jimma zone, southwest Ethiopia, 2016. (DOCX)

S5 Table KMO and Bartlett’s Test for wealth index items. (DOCX)

S6 Table Communalities for wealth index items. (DOCX)

S7 Table Total variance explained for wealth index items. (DOCX)

S1 Text Survey questionnaire in English. (DOCX)

S2 Text Survey questionnaire in survey language (Affan Oromo). (DOCX)
