# Supplementary material for: Are we too far from being client centered?
Source: PLoS One. 2018 Oct 15;13(10):e0205681. doi: 10.1371/journal.pone.0205681 (PMC6188795; doi:10.1371/journal.pone.0205681)
Supplement: S3 Table — (DOCX) [file pone.0205681.s003.docx]

**S3 Table:** Participants’ response on perceived quality of institutional delivery services on health facility/structure dimensions in public health institutions of three districts of Jimma zone, southwest Ethiopia, 2016.

| **Items** | 1 | 2 | 3 | 4 | 5 |
| --- | --- | --- | --- | --- | --- |
|  | No. (%) | No. (%) | No. (%) | No. (%) | No. (%) |
| **Health facility/structure dimension** |  |  |  |  |  |
| Health staffs suitability to treat women health problems | 2(.5) | 59(14.4) | 15(3.6) | 269(65.5) | 66(16.1) |
| Adequacy of delivery room | 11(2.7) | 144(35.0) | 14(3.4) | 188(45.7) | 54(13.1) |
| Adequacy of water for women in the facility | 93(22.6) | 179(43.6) | 16(3.9) | 87(21.2) | 36(8.8) |
| Cleanliness of the health facility | 6(1.5) | 151(36.7) | 33(8.0) | 162(39.4) | 59(14.4) |
| Equipment suitability | 8(1.9) | 121(29.4) | 66(16.1) | 177(43.1) | 39(9.5) |
| Health staff adequacy | 6(1.5) | 63(15.3) | 32(7.8) | 237(57.7) | 73(17.8) |

Strongly disagree (1), Disagree (2), Neutral (3), Agree (4) and strongly agree (5)
