# Supplementary material for: Are we too far from being client centered?
Source: PLoS One. 2018 Oct 15;13(10):e0205681. doi: 10.1371/journal.pone.0205681 (PMC6188795; doi:10.1371/journal.pone.0205681)
Supplement: S4 Table — (DOCX) [file pone.0205681.s004.docx]

**S4 Table:** Participants’ response on perceived quality of institutional delivery services on health care delivery dimension in public health institutions of three districts of Jimma zone, southwest Ethiopia, 2016

| **Items** | **1** | **2** | **3** | **4** | **5** |
| --- | --- | --- | --- | --- | --- |
|  | **No. (%)** | **No. (%)** | **No. (%)** | **No. (%)** | **No. (%)** |
| **Health care delivery dimension** |  |  |  |  |  |
| Privacy during delivery | 12(2.9) | 35(8.5) | 15(3.6) | 191(46.5) | 158(38.4) |
| Needed drugs prescription | 1(.2) | 65(15.8) | 13(3.2) | 248(60.3) | 84(20.4) |
| Good drugs supply | 4(1.0) | 60(14.6) | 18(4.4) | 263(64.0) | 66(16.1) |
| Easy availability of drugs | 9(2.2) | 126(30.7) | 25(6.1) | 185(45.0) | 66(16.1) |

Strongly disagree (1), Disagree (2), Neutral (3), Agree (4) and strongly agree (5)
