# Supplementary material for: Are we too far from being client centered?
Source: PLoS One. 2018 Oct 15;13(10):e0205681. doi: 10.1371/journal.pone.0205681 (PMC6188795; doi:10.1371/journal.pone.0205681)
Supplement: S5 Table — (DOCX) [file pone.0205681.s005.docx]

**S5 Table:** KMO and Bartlett’s Test for wealth index items

| Kaiser-Meyer-Olkin Measure of Sampling Adequacy. | | .720 |
| --- | --- | --- |
| Bartlett's Test of Sphericity | Approx. Chi-Square | 926.692 |
|  | df | 66 |
|  | Sig. | .000 |

**S6 Table:** Communalities for wealth index items

| **Items** | **Initial** | **Extraction** |
| --- | --- | --- |
| Function television | 1.000 | .591 |
| Does the house hold has stove | 1.000 | .694 |
| Does the house hold has motorcycle | 1.000 | .723 |
| Does the house hold has cart or gari | 1.000 | .768 |
| Does the house hold has mobile phone | 1.000 | .552 |
| Does the house hold has oxen | 1.000 | .722 |
| Does the house hold has cows | 1.000 | .603 |
| Does the house hold has goats or sheep | 1.000 | .786 |
| Type of toilet | 1.000 | .642 |
| Roof material | 1.000 | .679 |
| Number of rooms | 1.000 | .670 |
| Agricultural land owner | 1.000 | .616 |

Extraction Method: Principal Component Analysis.

**S7 Table:** Total variance explained for wealth index items

| **Component** | **Initial Eigen values** | | | **Extraction Sums of Squared Loadings** | | | **Rotation Sums of Squared Loadings** | | |
| --- | --- | --- | --- | --- | --- | --- | --- | --- | --- |
|  | Total | % of Variance | Cumulative % | Total | % of Variance | Cumulative % | Total | % of Variance | Cumulative % |
| 1 | 2.943 | 24.529 | 24.529 | 2.943 | 24.529 | 24.529 | 2.155 | 17.957 | 17.957 |
| 2 | 1.806 | 15.053 | 39.582 | 1.806 | 15.053 | 39.582 | 1.781 | 14.844 | 32.801 |
| 3 | 1.317 | 10.973 | 50.554 | 1.317 | 10.973 | 50.554 | 1.553 | 12.939 | 45.740 |
| 4 | 1.126 | 9.387 | 59.941 | 1.126 | 9.387 | 59.941 | 1.354 | 11.283 | 57.023 |
| 5 | .853 | 7.105 | 67.046 | .853 | 7.105 | 67.046 | 1.203 | 10.023 | 67.046 |
| 6 | .769 | 6.410 | 73.456 |  |  |  |  |  |  |
| 7 | .686 | 5.713 | 79.169 |  |  |  |  |  |  |
| 8 | .612 | 5.098 | 84.266 |  |  |  |  |  |  |
| 9 | .568 | 4.730 | 88.996 |  |  |  |  |  |  |
| 10 | .501 | 4.177 | 93.173 |  |  |  |  |  |  |
| 11 | .466 | 3.884 | 97.057 |  |  |  |  |  |  |
| 12 | .353 | 2.943 | 100.000 |  |  |  |  |  |  |

Extraction Method: Principal Component Analysis.

**S8 Text:** Survey questionnaire in English

Date ---------------Signature of the data collector to certify the informed consent verbally -------

Exit interview for perceived quality of delivery services

Instruction for the interviewer

Write Tick or number, or statements or word of the interviewed mother in front of the question of space provided ____ of this space

Starting tim_________End time_________

Date of data collection----------------------------------------

Name of data collector--------------------------------------- signature--------------------

Name of supervisor------------------------------------------- signature--------------------

Questionnaire Code __________

Part I Socio demographic characteristics of the clients

| S. N | Questions | Choice Answers |  |
| --- | --- | --- | --- |
| 101 | Current age of mother | in years--------------- |  |
| 102 | Where is your place of residence? | 1.Rural  2.Urban |  |
| 103 | What is your marital status? | 1. single  2. Divorced  3. Widowed  4. Married  5. Separated |  |
| 104 | What is your religion? | 1.Orthodox  2.Muslim  3.Protestant  4.Catholic  5.Others, specify----------------- |  |
| 105 | To which ethnic group do you belong? | 1. Oromo  2. Amhara  3. Gurage  4. Dawero  5. Kefa  6. Others, specify-------------- |  |
| 106 | What is your occupation? | 1. House wife  2. Government Employee  3. Farmer  4. Merchant  5. Daily laborer  6. Others, specify------------- |  |
| 107 | What is educational level of the mother? | 1.Unable to read and write  2.Read and write  3.Primary education(1-8)  4.Secondary education(9-12)  5.College and above |  |
| 108 | How many hours /kilometers does it take to reach the health facility you delivered? | ------------------hours or  ------------------kilometers |  |
| 109 | What mode of transport you used to reach the health facility you delivered? | 1. On foot 2.Ambulance 3.Other vehicles 4.On hourse /mule back 5.Local stretcher 6.Other,specify------- |  |
| 110 | Family size | In number ------------------------------ |  |
| 111 | Did you have information about skilled institutional health care services? | 1.yes 2.No |  |
| 112 | If yes, What were your sources of information about skilled institutional health care services? | 1.Health extension workers  2.Health facility  3.Mass media  4.Women development army  5.Friends and family  6.Others specify_________ |  |
| 113 | Do you have exposure to mass media | 1.Yes 2.No |  |
| 114 | Do you have any of the following means of communication? | 1 Radio  2.Television  3.both television and radio  4.Others, specify----------------- |  |

Part II Obstetric factors

| 201 | What is the total number of pregnancies in your life time? | In numbers--------------- | Probe for abortions |
| --- | --- | --- | --- |
| 202 | What is the total number of live births? (parity) | In numbers ----------------------------- |  |
| 203 | Have you ever had history of abortion? | 1.Yes 2.No | If noQ205 |
| 204 | If yes how many times? | 1. One 2.Two 3.Three 4.More than three |  |
| 205 | Have you ever had history of still birth? | 1.Yes 2.No | If notQ207 |
| 206 | If yes how many times? | 1. One 2.Two 3.Three 4.More than three |  |
| 207 | Do you have any complication during delivery of the last child | 1. Yes 2. No | If not skip to Q209 |
| 208 | If yes for question number 207 what were the complications? | 1.Severe vaginal bleeding  2.Severe Head ache  3.Marked & fast weight gain  4.Prolonged labor  5.Retained placenta  6.Other, specify---------------- |  |
| 209 | Did you visit to health facility for ANC during your last pregnancy? | 1.Yes  2.No | If not skip to Q 215 |
| 210 | If you visit for ANC, number of visits | --------------------- |  |
| 211 | Where did you attend ANC follow up? | 1. Hospital  2. Health Center  3. Private clinic  4. NGO Clinic  5. other specify_____ |  |
| 212 | During ANC follow up did you get any information about place of delivery & delivery Complications? | 1.Yes  2.No | If not Q215 |
| 213 | If yes, what types of information?  (multiple answers are possible) | 1.Severe vaginal bleeding  2.Severe Head ache  3.Marked & fast weight gain  4.Delivery at health facility  5.Prolonged labor  6.Retained placenta  7.Other, specify------------------------ |  |
| 214 | Was your last pregnancy planned? | 1. Yes 2.No |  |
| 215 | Who decide the place where you gave last birth? | 1. Myself 2.My husband 3.Both of us   4.Other,specify------------------- |  |
| 216 | What method of delivery? | 1. Normal vaginal delivery 2. Cesarean section 3. Assisted vaginal delivery 4. Episiotomy |  |
| 217 | Sex of the health care provider who attended the delivery | 1. Male 2.Female |  |
| 218 | Why did you choose to deliver in Health facility? | 1.To get better services in health facilities  2.To get better outcomes from health facilities to me and my baby  3.Bad experience from past home delivery  4.I was informed to deliver in health facilities  5.The health facility closer to my home  6.Others, specify---------------------------- |  |
| 219 | In which public health institution do you gave birth? | 1. Health center 2. Hospital |  |
| 220 | How many hours do you expend during labour? | 1. < 6 hour 2. 6-12 hour 3. 12-24 hour 4. Above 24 hour |  |
| 221 | Outcome of last pregnancy | 1.Live birth  2.Still birth |  |

**Part III: Questions on perceived quality of institutional birth services**

|  |  | Strongly agree | Agree | Neutral | Disagree | Strongly disagree |
| --- | --- | --- | --- | --- | --- | --- |
| 301 | In your opinion, the number of health staff in the public health institution is **adequate.** | 5 | 4 | 3 | 2 | 1 |
| 302 | In your opinion, the health staffs in the health facility are **well suited** to treat women’s health problems. | 5 | 4 | 3 | 2 | 1 |
| 303 | In your opinion, the delivery room of the health facility is adequate for mothers. | 5 | 4 | 3 | 2 | 1 |
| 304 | In your opinion, the provision of clean drinking water for women in the health facility are **adequate** | 5 | 4 | 3 | 2 | 1 |
| 305 | In your opinion, the overall environment of the public health institution is **very clean.** | 5 | 4 | 3 | 2 | 1 |
| 306 | In your opinion, the equipment in the public health institution is **well suited** for detecting women’s health problems. | 5 | 4 | 3 | 2 | 1 |
| 307 | In your opinion, the distance from your home to the health facility is **near** | 5 | 4 | 3 | 2 | 1 |
| 308 | In your opinion, the health staff in the public health institution examines pregnant and postpartum **women well.** | 5 | 4 | 3 | 2 | 1 |
| 309 | The public health institution provided **very much privacy** during vaginal examination and delivery. | 5 | 4 | 3 | 2 | 1 |
| 310 | In your opinion, the health staffs in the public health institution prescribe the drugs that are **needed.** | 5 | 4 | 3 | 2 | 1 |
| 311 | In your opinion, the drugs supplied by the public health institution are **good**. | 5 | 4 | 3 | 2 | 1 |
| 312 | In your opinion, mothers can obtain drugs from the public health institution **easily.** | 5 | 4 | 3 | 2 | 1 |
| 313 | In your opinion, during delivery care procedures you feel necessary act from health staffs. | 5 | 4 | 3 | 2 | 1 |
| 314 | In your opinion, the information of danger signs of delivery and postpartum provided by health staff is **adequate.** | 5 | 4 | 3 | 2 | 1 |
| 315 | In your opinion, the health staffs in the public health institution are **very capable** of finding out what is wrong with the clients. | 5 | 4 | 3 | 2 | 1 |
| 316 | In your opinion, the health staffs in the public health institution are **very open** with mothers. | 5 | 4 | 3 | 2 | 1 |
| 317 | In your opinion, the health staffs in the public health institution are **very compassionate** towards the mothers. | 5 | 4 | 3 | 2 | 1 |
| 318 | In your opinion, the health staffs are **respectful** towards the mothers. | 5 | 4 | 3 | 2 | 1 |
| 319 | In your opinion, the time that the health staffs devote to their clients is **adequate.** | 5 | 4 | 3 | 2 | 1 |
| 320 | In your opinion, the health staffs in the public health institution are **very honest**. | 5 | 4 | 3 | 2 | 1 |

1. Household wealth index identification questionnaires’

| Part I- Households wealth. Now I will ask you about some fixed assets that your households have. | | | |
| --- | --- | --- | --- |
| 401 Does the household has any of the following properties.(circle) | | Yes(1) | No(0) |
|  | Functioning radio/tape | 1 | 0 |
|  | Functioning television | 1 | 0 |
|  | Stove(gas/kerosene/electric) | 1 | 0 |
|  | motorcycle | 1 | 0 |
|  | Cart/Gari | 1 | 0 |
|  | Watch(hand /wall) | 1 | 0 |
|  | Mobile phone | 1 | 0 |
|  | Sofa | 1 | 0 |
|  | Spring mattress | 1 | 0 |
|  | Foam/sponge mattress | 1 | 0 |
|  | generator | 1 | 0 |
|  | Tractor (agricultural) | 1 | 0 |
|  | Water pump | 1 | 0 |
| 402 | **Does the Household have the following animals?** | 1.yes 0.no | How many? |
|  | oxen |  |  |
|  | cows |  |  |
|  | Horse /mule |  |  |
|  | Goats /cheeps |  |  |
|  | Chicken |  |  |
|  | Donkey |  |  |

| 403 | What is the main source of drinking water for members of your household? | 1.piped water into dwelling  2.Piped water to yard/plot  3.Public tap/standpipe water  4.Borehole water  5.Protected Dug well  6.Unprotected Dug well  7.Protected spring Water  8.Unprotected spring Water  9.River  10.Others, SPECIFY _______________ |  |
| --- | --- | --- | --- |
| 404 | What kind of toilet facility do members of your household usually use? | 1. Pit latrine 2. Pit latrine with slab 3. Pit latrine without slab/Open pit 4. Ventilated improved pit latrine 5. No facility /bush/field 6. Other specify |  |
| 405 | Do you share this toilet facility with other households? | 1. Yes 0/ No | If no, skip to Q407 |
| 406 | If "yes for Q 405" how many households use this toilet facility? | No. Of households _______________ |  |
| 407 | Main material of the floor. Record observation | 1. Earth/sand 2. Dung 3. Wood 4. Cement 5. Other ,specify _____________ |  |
| 408 | Main material of the roof. Record observation | 1. No roof 2. Thatch/leaf 3. Corrugated iron /metal 4. Other, specify _____________ |  |
| 409 | Main material of the exterior walls. Record observation. | 1. Natural walls 2. No walls 3. Bamboo/wood with mud 4. Uncovered adobe 5. covered adobe 6. Plywood /Reused wood 7. Other specify___________________ |  |
| 410 | How many rooms do the household has? | No. of rooms ________ |  |
| 411 | How many bed rooms do the household has? | No. of rooms ________ |  |
| 412 | Does any member of this household own any agricultural land? | 1. Yes 0. No | If no, skip to Q 414 |
| 413 | If yes, How many (local units) of agricultural land do members of this household own? | Local units   1. Local units _______________ 2. Don't know |  |
| 414 | Does any member of this household have a bank or microfinance saving account? | 1. Yes 2. No |  |

**S9 Text:** Survey questionnaire in survey language (Affan Oromo)

**Guyyaa**________mallattoo nama oddeeffannoo funanuu waa’ee odeeffanichaa ibsuu isaaf

_____________________

**Gaafiilee yeroo tataajila fudhatanii bahan gaafatamu**

**Qajeelfama gaffii fi deebiif qopha’an**

Lakkoofsa yookiin jecha fuuladura gaaffii jiru itti maruun yookiin mallattoo gochuun agarsisi.

Yeroo itti jalqabame________________ yeroo itti dhume _____________

Guyyaa itti odeeffannoon sasaabame____________________

Maqaa nama odeeffannoo funanuu___________________ mallattoo _____________

Maqaa to’ataa ____________________________ mallattoo ______________

Lakkoofsa addaa gaaffii _____________

Kutaa 1 ffaa: Gaaffilee odeeffannoo waliigalaafi hawaasummaa ilaallatan.

| Lakk | Gaaffilee | Filannoowwan |  |
| --- | --- | --- | --- |
| 101 | Umriin kee meeqa? | ---------------- waggaa |  |
| 102 | Bakka jireenyaa | 1.Baadiyyaa  2.Magaalaa |  |
| 103 | Haala fuudhaa fi heerumaa | 1. Hin heerumne  2.Walhiikneerra  3. Abbaan warraa narraa du’e  4. Heerumeera  5.Adda baaneerra/waliin hin jiraannu |  |
| 104 | Amantaan kee maali? | 1. Ortodoksii  2. Muusiliima  3 Pirootestaantii  4.Kaatoolikii  5.Kan biraa, adda baasi ----------------- |  |
| 105 | Sab-lammiimkee maali | 1. Oromoo  2. Amaaraa  3. Guraagee  4. Dawuroo  5.Kafaa  6. Kan biraa, adda baasi--------------- |  |
| 106 | Hojiin kee maali? | 1.Haadha warraa  2. Hojjetaa dhaabbata mootummaa  3. Qotee bulaa  4. Daldalaa  5. Hojjetaa guyyaa/dafqaan bulaa  6. Kan biraa, adda baasi ------------- |  |
| 107 | Sadarkaan barumsaa hammami? | 1.Barreessuufi dubbisuu kan hin dandeenye  2. Barreessuufi dubbisuu kan dandeessu  3.Barumsa sadarkaa jalqabaa(1-8)  4.Sadarkaa lammaffaafi(9-12)  5.Koollejjii fi isaa oli |  |
| 108 | Gara dhaabbata fayyaa ati itti deesse kana ga’uuf kiiloomeetira/ sa’atii meeqa sitti fudhata? | ------------------sa’atii ykn  ------------------kiiloomeetira |  |
| 109 | Gara dhaabbata fayyaa ati itti deesse kana ga’uuf geejjiba akkamiin dhufte? | 1.miilaan 2.Ambulaansiidhaan 3.konkolaataa gosa biraa 4.Gaangee/Fardaan 5.Namaan baatamee 6.Kan biraa ,adda baasi------- |  |
| 110 | Baayyinni maatii keessanii meeqa? | --------------------------(lakkoofsaan) |  |
| 111 | Odeffannoo Tajaajila da’umsaa dhaabbata fayyaatti ogeessa fayyaan deeggaramuun kennamuu dhagesse bektaa? | 1. Eyyee 2. lakkii |  |
| 112 | Eeyye yoo ta’e, maddi odeeffannoo Tajaajila da’umsaa dhaabbata fayyaatti ogeessa fayyaan deeggaramuun kennamuu maal ture? | 1. Ogeettii Ekisteenshinii fayyaa  2. Dhaabbata fayyaa  3. Miidiyaalee hawaasaa  4. Gamta tokko shanee  5. Hiriyyootaafi maatiiwwan  6. Kan biraa, adda baasi_________ |  |
| 113 | Miidiyaalee adda addaa nii hordoftaa? | 1. Eyyee 2. lakkii |  |
| 114 | Wantoota odeeffannoo dabarsuuf  / argachuuf gargaaran kanneen keessaa maalfaa qabda? | 1. Raadiyoo  2. Televizhiinii  3. oomaa hin qabu  4.Kan biraa, adda baasi----------------- |  |

Kutaa 2ffaa. Gaaffii ogummaa deessissuun wal qabate.

| 201 | Hanga ammaatti waliigalatti yeroo meeqa ulfooftee jirta? | Lakkoofsaan_________  (yoo jira ta’e kan osoo hingahiin sirraa bahe fi kan du’ee dhalate dabalatee) |  |
| --- | --- | --- | --- |
| 202 | Hanga ammaatti daa’ima meeqa deesseetta? | Lakkoofsaan________________ |  |
| 203 | Kana dura ulfi sirraa ba’ee turee? | 1.Eyyee 2.Lakkii |  |
| 204 | Gaaffii 203’f yoo deebiin kee Eyyee ta’e, ala meeqa? | 1. Tokko 2.Lama 3.Sadii 4.Sadii oli |  |
| 205 | Kana dura daa’ima lubbuu hin qabne deessee turtee? | 1.Eyyee 2.Lakkii |  |
| 206 | Gaaffii 205’f yoo deebiin kee Eyyee ta’e, ala meeqa? | 1. Tokko 2.Lama 3.Sadii 4.Sadii oli |  |
| 207 | Daa’ima kee kan dhuma irratti deesse kana yeroo deesse rakkoon da’umsaan wal qabate simudatee turee? | 1. Eyyee 2. lakki |  |
| 208 | Yoo deebiin kee gaaffii 207 eyyee ta’e, rakkoolee kamfaatu si mudate? | 1. qaama hormaataan dhiiqni dhangala’uu.  2. bowwuu mataa cimaa.  3. ulfattinni qaamaa saffisaan dabaluu  4. Da’umsa mana yaalaatti taasisuu.  5. Miixuu/ciniinsuu sa’a dheeraa  6. Obbaattii/ofkaltiin ba’uu dhabuu.  7. kan biraa ibsi---------------------- |  |
| 209 | Da’umsa kee kan xumuraa kana irratti hordoffii kammiinuu taasifteettaa? | 1. eeyyee 2. lakki |  |
| 210 | Sababni ati gara mana yaalaa deemteef hordoffii da’umsa duraaf ta’e, si’a meeqa deemte? | Lakkoofsaan______________ |  |
| 211 | Hordoffii da’umsa duraa eessatti taasifte? | 1. hoospitaala  2. buufata fayyaa  3.kilinika dhunfaa  4. Kilinikaa mitmotumma  5. kan biraa, ibsi________________ |  |
| 212 | Yeroo Hordoffii da’umsa duraa taasifte odeeffannoo iddoo da’umsaa filachuu fi balaa hamaa yeroo da’umsaa argatteettaa? | 1. Eyyee 2. Lakki |  |
| 213 | Yoo eeyyee jette odeeffannoo akkamii?  (deebii tokkoo ol kennuun ni danda’ama) | 1. qaama hormaataan dhiiqni dhangala’uu.  2. bowwuu mataa cimaa.  3. ulfattinni qaamaa saffisaan dabaluu  4. Da’umsa mana yaalaatti taasisuu.  5. Miixuu/ciniinsuu sa’a dheeraa  6. Obbaattii/ofkaltiin ba’uu dhabuu.  7. kan biraa ibsi. |  |
| 214 | Ulffii kee inni dhuma kanaa karoora kee turee? | 1. Eyyee  2. Lakki |  |
| 215 | Daa’ima kee dhumaa eessatti da’uu akka qabdu kan murteessee eenyu ture? | 1. Anuma mataa koo  2.Abbaawarraa koo  3. Lamaan keenya  4. Ekisteenshinii fayyaa  5.kan biraa, adda baasi------------------------ |  |
| 216 | Mala kamiin deessee | 1.Gadameessaan  2.Garaa baqaqsaan  3.Mees haadhaan gargaara manii na deessisanii  4.Qaama saalaa kutanii/baqaqsanii hodhuun |  |
| 217 | Ogeessi sideessise/te dhiira moo dhalaa dha? | 1. Dhiira 2. Dhalaa |  |
| 218 | Dhabbata fayyaa keessaatti dahuuf maliif filtee? | 1.Tajaajila foyya’aa argachuuf  2. Bu’aa foyya’aa ofii kooti fi mucaa kottif argachhuf.  3.Muxxannoo badaa/gadhee/ manatti dhaluu yeroo darbee  4.Dhabbata fayyatti akkan dhaluu natti himameeti  5. Dhabbanni fayyaa mana kotti dhihoo jira.  6. Kan biro yoo jiratee ibsi-------------------- |  |
| 219 | Dhabbata fayyatti yoo deessee, dhabbataa fayyaa kamiitti deessee? | 1.Bufata fayyaa  2.Hospitaala |  |
| 220 | Sa’atii cinsuun sirra ture | 1. sa’a 6 gadii  2. sa’a 6- 12:00  3. sa’a 12- 24:00  4. sa’a 24 ol |  |
| 221 | Xumura/rawwii ulfaa isa dhihoo | 1. Lubbuu qaba.  2.kan du’ee/lubbu kan hin qabne |  |

Kutaa 3 ffaa: Gaaffilee ilaalchaa qulqullina tajaajila da’umsaa dhaabbata fayyaa irratti dhiyaatan.

| Lakk | **Dhaabbata fayyaa** | | | | | |
| --- | --- | --- | --- | --- | --- | --- |
|  | Gaaffilee | Sirriitti ittin walii gala | Ittin waliigala | yaada hin qabu | itti walii hin galu | sirriitti itti walii hin galu |
| 301 | Akka yaada keetti, baayyinni hojjettoota dhaabbata fayyaa keessa hojjetanii ga’aa dha. | 5. | 4. | 3. | 2. | 1. |
| 302 | Akka yaada keetti, baayyinni hojjettoota dhaabbata fayyaa ati itti fayyadamte keessa hojjetanii rakkoolee haadholii yaaluuf mijatoo dha. | 5. | 4. | 3. | 2. | 1. |
| 303 | Akka yaada keetti, bakki turanii dabaree eegan, kutaan sakatta’insaa ykn qorannoo yaalaa fi kutaaleen kan biroon dhaabbata fayyaa rakkoo haadholii furuuf ga’aadha. | 5. | 4. | 3. | 2. | 1. |
| 304 | Akka yaada keetti, dhiyeessi bishaan dhugaatii qulqulluu, bakki harka itti dhiqatanii fi manneen boolii ykn Fincaanii dhaabbata fayyaa keessatti argaman haadholiidhaaf ga’aadha. | 5. | 4. | 3. | 2. | 1. |
| 305 | Akka yaada keetti, walumaagallli naannoo dhaabbata fayyaa kanaa baayyeee qulqulluudha. | 5. | 4. | 3. | 2. | 1. |
| 306 | Akka yaada keetti, meeshaleen dhaabbata fayyaa kanaa keesatti argaman rakkoo haadholii sirriitti adda baasuuf kan sadarkaan isaanii eegamedha. | 5. | 4. | 3. | 2. | 1. |
| 307 | Fageenyi mana kee fi dhaabbata fayyaa kana gidduu jiru baayyee fagoodha. | 5. | 4. | 3. | 2. | 1. |
| 308 | Akka yaada keetti, hojjettoonni dhaabbata fayyaa kanaa haadholii yeroo ulfaafi da’umsa booda sirriitti qoratu/sakatta’u. | 5. | 4. | 3. | 2. | 1. |
| 309 | Dhaabbanni fayyaa kanaa sakatta’insa qaama hormaataafi tajaajila da’umsaa bakka mijataa fi namni nama arguu hin dandeenyetti kenna. | 5. | 4. | 3. | 2. | 1. |
| 310 | Akka yaada keetti, hojjettoonni dhaabbata fayyaa kanaa qorichoota barbaachisan ajaju/barreessu. | 5. | 4. | 3. | 2. | 1. |
| 311 | Akka yaada keetti, qorichoonni dhaabbata fayyaa kanaan kennaman gaariidha/baroodha. | 5. | 4. | 3. | 2. | 1. |
| 312 | Akka yaada keetti, haadholiin qoricha haaluma salphaan dhaabbata fayyaa kanarraa argachuu danda’u. | 5. | 4. | 3. | 2. | 1. |
| 313 | Sababa duraa duuba gochaalee hin barbaachisne yeroo da’umsaatiin miirrii nuffisiisaa fiqaaneffacuu natti dhagahameera. | 5. | 4. | 3. | 2. | 1. |
| 314 | Akka yaada keetti, odeeffannoo/hubannoo mallattoo balaa cimaa yeroo da’umsaa fi da’umsa boodaa hojjettoonni fayyaa kennan ga’aadha. | 5. | 4. | 3. | 2. | 1. |
| 315 | Akka yaada keetti, hojjetoonni fayyaaa dhaabbata fayyaa kana keessa hojjetan,rakkoo fayyadamtootaa sirriitti adda baasuu nii danda’u. | 5. | 4. | 3. | 2. | 1. |
| 316 | Akka yaada keetti, hojjettoonni dhaabbata fayyaa kana keessa hojjetan haadholiififtoomina baayyee dansaa/gaarii qabu. | 5. | 4. | 3. | 2. | 1. |
| 317 | Akka yaada keetti, hojjettoonni dhaabbata fayyaa kana keessa hojjetan haadholiif baayyee dhimmamoodha. | 5. | 4. | 3. | 2. | 1. |
| 318 | Akka yaada keetti, hojjettoonni dhaabbata fayyaa kanaa haadholiif sirriitti ni kabaju. | 5. | 4. | 3. | 2. | 1. |
| 319 | Akka yaada keetti, yeroon hojjettoonni fayyaa haadholiif qoodan ga’aadha. | 5. | 4. | 3. | 2. | 1. |
| 320 | Akka yaada keetti, hojjetoonni fayyaaa dhaabbata fayyaa kana keessa hojjetan sirriitti amanamoodha. | 5. | 4. | 3. | 2. | 1. |

Gaaffilee sassaabbii ragaa qabeenyaa Manneenii

| Kutaa 1- Ragaa qabeenyaa. Armaan gaditti meshaalee mana keessan keessatti argamanu isinan gaafadha | | | |
| --- | --- | --- | --- |
| 401 Mana kana keessa meshaalee armaan gadii kessaa kamtu jiraa ? yoo jiraatee (1) tti yoo hin jirre (0) tti mari | | Eyyee(1) | Hinjiru (0) |
|  | Raadiyo/CD/”tape recordarii’ hojjetu | 1 | 0 |
|  | Televisi’onii hojjetu | 1 | 0 |
|  | Stoovii/gaazii/ ibsaa elektrikaa | 1 | 0 |
|  | motorsaaykilii | 1 | 0 |
|  | Gaarii fardaa | 1 | 0 |
|  | Sa’aatii girgiddaa | 1 | 0 |
|  | Mobaayilii | 1 | 0 |
|  | ‘Soofaa’ | 1 | 0 |
|  | Firaashii spoonjii | 1 | 0 |
|  | Firaashii cidii | 1 | 0 |
|  | Genereetarii | 1 | 0 |
|  | Tiraaktara Qonnaa | 1 | 0 |
| 402 | Manni kun bineelda manaa armaan gadii qabaa? | 1.eyyee 0.hinqabu | Meeqa? |
|  | sangaa | 1.eyyee 0.hinqabu |  |
|  | Sa’a | 1.eyyee 0.hinqabu |  |
|  | Farad/gaangee | 1.eyyee 0.hinqabu |  |
|  | Hoolaa/ reetii | 1.eyyee 0.hinqabu |  |
|  | Reetii | 1.eyyee 0.hinqabu |  |
|  | Harree | 1.eyyee 0.hinqabu |  |

| 403 | Maatiin keessan bishaan dhugaatii eessaa argataa? | 1. bishaan boollaa itti ijaarame  2. bishaan boollaa itti hin ijaaramin  3. burqituu itti ijaarame  4.burqituu itti hin ijaaramin  5.Bishaan Biirii  6. bishaan bollaa paampii kan uummataa  6.Bishaan lagaa yaa’u  7.Ujummoo/sarara bishaanii dallaa keessaa  8. Ujummoo/sarara bishaanii dallaan alaa  Kan biro,ibsi ___________ |  |
| --- | --- | --- | --- |
| 404 | Maatiin keessan mana fincaanii akkamiitti fayyadama? | Boolla qotamaatti  Boolla fincaanii dahannaa qabu  Boolla fincaanii dahannaa hin qabne  Mana fincaanii sadarkaa isaa eeggate  Dirree,ykn bakkee irratti  Kan biraa,ibsi____________ |  |
| 405 | Mana fincaanii kana maatiin kan biraa isinwaliin nifayyadama? | Eeyyee 0/ Lakki | Yoo lakkii ta’e gara gaaffii 407 tti darbi |
| 406 | Yoo deebiin 405 eeyyee ta’e Baayyinni Abbaa warraa isin waliin itti fayyadamanii meeqa? | Baayyina abbaa warraa_______________ |  |
| 407 | Hundeen lafa mana kanaa maalii?.Ilaalii /daawwadhu mirkaneessi. | Biyyoo/ lafa  Dikee /compostii  Muka  Simintoo  Kan biro ________________ |  |
| 408 | Ijoon (Uwwisi)) mana kanaa maali?  Ilaalii/daawwadhuu mirkaneessi. | Uwwisa hin qabu  Citaa ykn baala  Sibiila qorqorroo  Kan biraa___________________ |  |
| 409 | Duppon ykn Gidgiddaan mana kanaa maal irraa tolfame?  Ilaalii/daawwadhuu mirkaneessi. | Natural walls  Keenyan hin qabu  Mukaafi biyoo ykn Dhoqqee  Suphee ykn shakilaa duudaa hin ta’in  Bilookeetii ykn Shakilaa duudaa  Muka ykn xawulaa hin dulloomne (yeroo birraaf kan fayyadu)  Kan biraa___________________ |  |
| 410 | Manni keessan kun kutaa meeqa qaba? | Baayyina kutaa________ |  |
| 411 | Maatii keessan lafaqotisaa hagam qaba ? | Safartuu naannoo(hektaara) ---------------  2. Hin beeku |  |
| 412 | Maatii keessan keessaa namni accountii baankii ykn baankii qusanoo fayyadamu jiraa? | Eeyye  Hinjiru |  |
