# Supplementary material for: Are we too far from being client centered?
Source: PLoS One. 2018 Oct 15;13(10):e0205681. doi: 10.1371/journal.pone.0205681 (PMC6188795; doi:10.1371/journal.pone.0205681)
Supplement: S6 Table — (DOCX) [file pone.0205681.s006.docx]

**S6 Table:** Communalities for wealth index items

| **Items** | **Initial** | **Extraction** |
| --- | --- | --- |
| Function television | 1.000 | .591 |
| Does the house hold has stove | 1.000 | .694 |
| Does the house hold has motorcycle | 1.000 | .723 |
| Does the house hold has cart or gari | 1.000 | .768 |
| Does the house hold has mobile phone | 1.000 | .552 |
| Does the house hold has oxen | 1.000 | .722 |
| Does the house hold has cows | 1.000 | .603 |
| Does the house hold has goats or sheep | 1.000 | .786 |
| Type of toilet | 1.000 | .642 |
| Roof material | 1.000 | .679 |
| Number of rooms | 1.000 | .670 |
| Agricultural land owner | 1.000 | .616 |

Extraction Method: Principal Component Analysis.
