# Supplementary material for: Are we too far from being client centered?
Source: PLoS One. 2018 Oct 15;13(10):e0205681. doi: 10.1371/journal.pone.0205681 (PMC6188795; doi:10.1371/journal.pone.0205681)
Supplement: S7 Table — (DOCX) [file pone.0205681.s007.docx]

**S7 Table:** Total variance explained for wealth index items

| **Component** | **Initial Eigen values** | | | **Extraction Sums of Squared Loadings** | | | **Rotation Sums of Squared Loadings** | | |
| --- | --- | --- | --- | --- | --- | --- | --- | --- | --- |
|  | Total | % of Variance | Cumulative % | Total | % of Variance | Cumulative % | Total | % of Variance | Cumulative % |
| 1 | 2.943 | 24.529 | 24.529 | 2.943 | 24.529 | 24.529 | 2.155 | 17.957 | 17.957 |
| 2 | 1.806 | 15.053 | 39.582 | 1.806 | 15.053 | 39.582 | 1.781 | 14.844 | 32.801 |
| 3 | 1.317 | 10.973 | 50.554 | 1.317 | 10.973 | 50.554 | 1.553 | 12.939 | 45.740 |
| 4 | 1.126 | 9.387 | 59.941 | 1.126 | 9.387 | 59.941 | 1.354 | 11.283 | 57.023 |
| 5 | .853 | 7.105 | 67.046 | .853 | 7.105 | 67.046 | 1.203 | 10.023 | 67.046 |
| 6 | .769 | 6.410 | 73.456 |  |  |  |  |  |  |
| 7 | .686 | 5.713 | 79.169 |  |  |  |  |  |  |
| 8 | .612 | 5.098 | 84.266 |  |  |  |  |  |  |
| 9 | .568 | 4.730 | 88.996 |  |  |  |  |  |  |
| 10 | .501 | 4.177 | 93.173 |  |  |  |  |  |  |
| 11 | .466 | 3.884 | 97.057 |  |  |  |  |  |  |
| 12 | .353 | 2.943 | 100.000 |  |  |  |  |  |  |

Extraction Method: Principal Component Analysis.
