# Supplementary material for: Are we too far from being client centered?
Source: PLoS One. 2018 Oct 15;13(10):e0205681. doi: 10.1371/journal.pone.0205681 (PMC6188795; doi:10.1371/journal.pone.0205681)
Supplement: S1 Text — (DOCX) [file pone.0205681.s008.docx]

**S1 Text:** Survey questionnaire in English

Date ---------------Signature of the data collector to certify the informed consent verbally -------

Exit interview for perceived quality of delivery services

Instruction for the interviewer

Write Tick or number, or statements or word of the interviewed mother in front of the question of space provided ____ of this space

Starting time _________End time_________

Date of data collection----------------------------------------

Name of data collector--------------------------------------- signature--------------------

Name of supervisor------------------------------------------- signature--------------------

Questionnaire Code __________

Part I Socio demographic characteristics of the clients

| S. N | Questions | Choice Answers |  |
| --- | --- | --- | --- |
| 101 | Current age of mother | in years--------------- |  |
| 102 | Where is your place of residence? | 1.Rural  2.Urban |  |
| 103 | What is your marital status? | 1. single  2. Divorced  3. Widowed  4. Married  5. Separated |  |
| 104 | What is your religion? | 1.Orthodox  2.Muslim  3.Protestant  4.Catholic  5.Others, specify----------------- |  |
| 105 | To which ethnic group do you belong? | 1. Oromo  2. Amhara  3. Gurage  4. Dawero  5. Kefa  6. Others, specify-------------- |  |
| 106 | What is your occupation? | 1. House wife  2. Government Employee  3. Farmer  4. Merchant  5. Daily laborer  6. Others, specify------------- |  |
| 107 | What is educational level of the mother? | 1.Unable to read and write  2.Read and write  3.Primary education(1-8)  4.Secondary education(9-12)  5.College and above |  |
| 108 | How many hours /kilometers does it take to reach the health facility you delivered? | ------------------hours or  ------------------kilometers |  |
| 109 | What mode of transport you used to reach the health facility you delivered? | 1. On foot 2.Ambulance 3.Other vehicles 4.On hourse /mule back 5.Local stretcher 6.Other,specify------- |  |
| 110 | Family size | In number ------------------------------ |  |
| 111 | Did you have information about skilled institutional health care services? | 1.yes 2.No |  |
| 112 | If yes, What were your sources of information about skilled institutional health care services? | 1.Health extension workers  2.Health facility  3.Mass media  4.Women development army  5.Friends and family  6.Others specify_________ |  |
| 113 | Do you have exposure to mass media | 1.Yes 2.No |  |
| 114 | Do you have any of the following means of communication? | 1 Radio  2.Television  3.both television and radio  4.Others, specify----------------- |  |

Part II Obstetric factors

| 201 | What is the total number of pregnancies in your life time? | In numbers--------------- | Probe for abortions |
| --- | --- | --- | --- |
| 202 | What is the total number of live births? (parity) | In numbers ----------------------------- |  |
| 203 | Have you ever had history of abortion? | 1.Yes 2.No | If noQ205 |
| 204 | If yes how many times? | 1. One 2.Two 3.Three 4.More than three |  |
| 205 | Have you ever had history of still birth? | 1.Yes 2.No | If notQ207 |
| 206 | If yes how many times? | 1. One 2.Two 3.Three 4.More than three |  |
| 207 | Do you have any complication during delivery of the last child | 1. Yes 2. No | If not skip to Q209 |
| 208 | If yes for question number 207 what were the complications? | 1.Severe vaginal bleeding  2.Severe Head ache  3.Marked & fast weight gain  4.Prolonged labor  5.Retained placenta  6.Other, specify---------------- |  |
| 209 | Did you visit to health facility for ANC during your last pregnancy? | 1.Yes  2.No | If not skip to Q 215 |
| 210 | If you visit for ANC, number of visits | --------------------- |  |
| 211 | Where did you attend ANC follow up? | 1. Hospital  2. Health Center  3. Private clinic  4. NGO Clinic  5. other specify_____ |  |
| 212 | During ANC follow up did you get any information about place of delivery & delivery Complications? | 1.Yes  2.No | If not Q215 |
| 213 | If yes, what types of information?  (multiple answers are possible) | 1.Severe vaginal bleeding  2.Severe Head ache  3.Marked & fast weight gain  4.Delivery at health facility  5.Prolonged labor  6.Retained placenta  7.Other, specify------------------------ |  |
| 214 | Was your last pregnancy planned? | 1. Yes 2.No |  |
| 215 | Who decide the place where you gave last birth? | 1. Myself 2.My husband 3.Both of us   4.Other,specify------------------- |  |
| 216 | What method of delivery? | 1. Normal vaginal delivery 2. Cesarean section 3. Assisted vaginal delivery 4. Episiotomy |  |
| 217 | Sex of the health care provider who attended the delivery | 1. Male 2.Female |  |
| 218 | Why did you choose to deliver in Health facility? | 1.To get better services in health facilities  2.To get better outcomes from health facilities to me and my baby  3.Bad experience from past home delivery  4.I was informed to deliver in health facilities  5.The health facility closer to my home  6.Others, specify---------------------------- |  |
| 219 | In which public health institution do you gave birth? | 1. Health center 2. Hospital |  |
| 220 | How many hours do you expend during labour? | 1. < 6 hour 2. 6-12 hour 3. 12-24 hour 4. Above 24 hour |  |
| 221 | Outcome of last pregnancy | 1.Live birth  2.Still birth |  |

**Part III: Questions on perceived quality of institutional birth services**

|  |  | Strongly agree | Agree | Neutral | Disagree | Strongly disagree |
| --- | --- | --- | --- | --- | --- | --- |
| 301 | In your opinion, the number of health staff in the public health institution is **adequate.** | 5 | 4 | 3 | 2 | 1 |
| 302 | In your opinion, the health staffs in the health facility are **well suited** to treat women’s health problems. | 5 | 4 | 3 | 2 | 1 |
| 303 | In your opinion, the delivery room of the health facility is adequate for mothers. | 5 | 4 | 3 | 2 | 1 |
| 304 | In your opinion, the provision of clean drinking water for women in the health facility are **adequate** | 5 | 4 | 3 | 2 | 1 |
| 305 | In your opinion, the overall environment of the public health institution is **very clean.** | 5 | 4 | 3 | 2 | 1 |
| 306 | In your opinion, the equipment in the public health institution is **well suited** for detecting women’s health problems. | 5 | 4 | 3 | 2 | 1 |
| 307 | In your opinion, the distance from your home to the health facility is **near** | 5 | 4 | 3 | 2 | 1 |
| 308 | In your opinion, the health staff in the public health institution examines pregnant and postpartum **women well.** | 5 | 4 | 3 | 2 | 1 |
| 309 | The public health institution provided **very much privacy** during vaginal examination and delivery. | 5 | 4 | 3 | 2 | 1 |
| 310 | In your opinion, the health staffs in the public health institution prescribe the drugs that are **needed.** | 5 | 4 | 3 | 2 | 1 |
| 311 | In your opinion, the drugs supplied by the public health institution are **good**. | 5 | 4 | 3 | 2 | 1 |
| 312 | In your opinion, mothers can obtain drugs from the public health institution **easily.** | 5 | 4 | 3 | 2 | 1 |
| 313 | In your opinion, during delivery care procedures you feel necessary act from health staffs. | 5 | 4 | 3 | 2 | 1 |
| 314 | In your opinion, the information of danger signs of delivery and postpartum provided by health staff is **adequate.** | 5 | 4 | 3 | 2 | 1 |
| 315 | In your opinion, the health staffs in the public health institution are **very capable** of finding out what is wrong with the clients. | 5 | 4 | 3 | 2 | 1 |
| 316 | In your opinion, the health staffs in the public health institution are **very open** with mothers. | 5 | 4 | 3 | 2 | 1 |
| 317 | In your opinion, the health staffs in the public health institution are **very compassionate** towards the mothers. | 5 | 4 | 3 | 2 | 1 |
| 318 | In your opinion, the health staffs are **respectful** towards the mothers. | 5 | 4 | 3 | 2 | 1 |
| 319 | In your opinion, the time that the health staffs devote to their clients is **adequate.** | 5 | 4 | 3 | 2 | 1 |
| 320 | In your opinion, the health staffs in the public health institution are **very honest**. | 5 | 4 | 3 | 2 | 1 |

1. Household wealth index identification questionnaires’

| Part I- Households wealth. Now I will ask you about some fixed assets that your households have. | | | |
| --- | --- | --- | --- |
| 401 Does the household has any of the following properties.(circle) | | Yes(1) | No(0) |
|  | Functioning radio/tape | 1 | 0 |
|  | Functioning television | 1 | 0 |
|  | Stove(gas/kerosene/electric) | 1 | 0 |
|  | motorcycle | 1 | 0 |
|  | Cart/Gari | 1 | 0 |
|  | Watch(hand /wall) | 1 | 0 |
|  | Mobile phone | 1 | 0 |
|  | Sofa | 1 | 0 |
|  | Spring mattress | 1 | 0 |
|  | Foam/sponge mattress | 1 | 0 |
|  | generator | 1 | 0 |
|  | Tractor (agricultural) | 1 | 0 |
|  | Water pump | 1 | 0 |
| 402 | **Does the Household have the following animals?** | 1.yes 0.no | How many? |
|  | oxen |  |  |
|  | cows |  |  |
|  | Horse /mule |  |  |
|  | Goats /cheeps |  |  |
|  | Chicken |  |  |
|  | Donkey |  |  |

| 403 | What is the main source of drinking water for members of your household? | 1.piped water into dwelling  2.Piped water to yard/plot  3.Public tap/standpipe water  4.Borehole water  5.Protected Dug well  6.Unprotected Dug well  7.Protected spring Water  8.Unprotected spring Water  9.River  10.Others, SPECIFY _______________ |  |
| --- | --- | --- | --- |
| 404 | What kind of toilet facility do members of your household usually use? | 1. Pit latrine 2. Pit latrine with slab 3. Pit latrine without slab/Open pit 4. Ventilated improved pit latrine 5. No facility /bush/field 6. Other specify |  |
| 405 | Do you share this toilet facility with other households? | 1. Yes 0/ No | If no, skip to Q407 |
| 406 | If "yes for Q 405" how many households use this toilet facility? | No. Of households _______________ |  |
| 407 | Main material of the floor. Record observation | 1. Earth/sand 2. Dung 3. Wood 4. Cement 5. Other ,specify _____________ |  |
| 408 | Main material of the roof. Record observation | 1. No roof 2. Thatch/leaf 3. Corrugated iron /metal 4. Other, specify _____________ |  |
| 409 | Main material of the exterior walls. Record observation. | 1. Natural walls 2. No walls 3. Bamboo/wood with mud 4. Uncovered adobe 5. covered adobe 6. Plywood /Reused wood 7. Other specify___________________ |  |
| 410 | How many rooms do the household has? | No. of rooms ________ |  |
| 411 | How many bed rooms do the household has? | No. of rooms ________ |  |
| 412 | Does any member of this household own any agricultural land? | 1. Yes 0. No | If no, skip to Q 414 |
| 413 | If yes, How many (local units) of agricultural land do members of this household own? | Local units   1. Local units _______________ 2. Don't know |  |
| 414 | Does any member of this household have a bank or microfinance saving account? | 1. Yes 2. No |  |
