# Supplementary material for: Are we too far from being client centered?
Source: PLoS One. 2018 Oct 15;13(10):e0205681. doi: 10.1371/journal.pone.0205681 (PMC6188795; doi:10.1371/journal.pone.0205681)
Supplement: S2 Text — (DOCX) [file pone.0205681.s009.docx]

**S2 Text:** Survey questionnaire in survey language (Affan Oromo)

**Guyyaa**________mallattoo nama oddeeffannoo funanuu waa’ee odeeffanichaa ibsuu isaaf

_____________________

**Gaafiilee yeroo tataajila fudhatanii bahan gaafatamu**

**Qajeelfama gaffii fi deebiif qopha’an**

Lakkoofsa yookiin jecha fuuladura gaaffii jiru itti maruun yookiin mallattoo gochuun agarsisi.

Yeroo itti jalqabame________________ yeroo itti dhume _____________

Guyyaa itti odeeffannoon sasaabame____________________

Maqaa nama odeeffannoo funanuu___________________ mallattoo _____________

Maqaa to’ataa ____________________________ mallattoo ______________

Lakkoofsa addaa gaaffii _____________

Kutaa 1 ffaa: Gaaffilee odeeffannoo waliigalaafi hawaasummaa ilaallatan.

| Lakk | Gaaffilee | Filannoowwan |  |
| --- | --- | --- | --- |
| 101 | Umriin kee meeqa? | ---------------- waggaa |  |
| 102 | Bakka jireenyaa | 1.Baadiyyaa  2.Magaalaa |  |
| 103 | Haala fuudhaa fi heerumaa | 1. Hin heerumne  2.Walhiikneerra  3. Abbaan warraa narraa du’e  4. Heerumeera  5.Adda baaneerra/waliin hin jiraannu |  |
| 104 | Amantaan kee maali? | 1. Ortodoksii  2. Muusiliima  3 Pirootestaantii  4.Kaatoolikii  5.Kan biraa, adda baasi ----------------- |  |
| 105 | Sab-lammiimkee maali | 1. Oromoo  2. Amaaraa  3. Guraagee  4. Dawuroo  5.Kafaa  6. Kan biraa, adda baasi--------------- |  |
| 106 | Hojiin kee maali? | 1.Haadha warraa  2. Hojjetaa dhaabbata mootummaa  3. Qotee bulaa  4. Daldalaa  5. Hojjetaa guyyaa/dafqaan bulaa  6. Kan biraa, adda baasi ------------- |  |
| 107 | Sadarkaan barumsaa hammami? | 1.Barreessuufi dubbisuu kan hin dandeenye  2. Barreessuufi dubbisuu kan dandeessu  3.Barumsa sadarkaa jalqabaa(1-8)  4.Sadarkaa lammaffaafi(9-12)  5.Koollejjii fi isaa oli |  |
| 108 | Gara dhaabbata fayyaa ati itti deesse kana ga’uuf kiiloomeetira/ sa’atii meeqa sitti fudhata? | ------------------sa’atii ykn  ------------------kiiloomeetira |  |
| 109 | Gara dhaabbata fayyaa ati itti deesse kana ga’uuf geejjiba akkamiin dhufte? | 1.miilaan 2.Ambulaansiidhaan 3.konkolaataa gosa biraa 4.Gaangee/Fardaan 5.Namaan baatamee 6.Kan biraa ,adda baasi------- |  |
| 110 | Baayyinni maatii keessanii meeqa? | --------------------------(lakkoofsaan) |  |
| 111 | Odeffannoo Tajaajila da’umsaa dhaabbata fayyaatti ogeessa fayyaan deeggaramuun kennamuu dhagesse bektaa? | 1. Eyyee 2. lakkii |  |
| 112 | Eeyye yoo ta’e, maddi odeeffannoo Tajaajila da’umsaa dhaabbata fayyaatti ogeessa fayyaan deeggaramuun kennamuu maal ture? | 1. Ogeettii Ekisteenshinii fayyaa  2. Dhaabbata fayyaa  3. Miidiyaalee hawaasaa  4. Gamta tokko shanee  5. Hiriyyootaafi maatiiwwan  6. Kan biraa, adda baasi_________ |  |
| 113 | Miidiyaalee adda addaa nii hordoftaa? | 1. Eyyee 2. lakkii |  |
| 114 | Wantoota odeeffannoo dabarsuuf  / argachuuf gargaaran kanneen keessaa maalfaa qabda? | 1. Raadiyoo  2. Televizhiinii  3. oomaa hin qabu  4.Kan biraa, adda baasi----------------- |  |

Kutaa 2ffaa. Gaaffii ogummaa deessissuun wal qabate.

| 201 | Hanga ammaatti waliigalatti yeroo meeqa ulfooftee jirta? | Lakkoofsaan_________  (yoo jira ta’e kan osoo hingahiin sirraa bahe fi kan du’ee dhalate dabalatee) |  |
| --- | --- | --- | --- |
| 202 | Hanga ammaatti daa’ima meeqa deesseetta? | Lakkoofsaan________________ |  |
| 203 | Kana dura ulfi sirraa ba’ee turee? | 1.Eyyee 2.Lakkii |  |
| 204 | Gaaffii 203’f yoo deebiin kee Eyyee ta’e, ala meeqa? | 1. Tokko 2.Lama 3.Sadii 4.Sadii oli |  |
| 205 | Kana dura daa’ima lubbuu hin qabne deessee turtee? | 1.Eyyee 2.Lakkii |  |
| 206 | Gaaffii 205’f yoo deebiin kee Eyyee ta’e, ala meeqa? | 1. Tokko 2.Lama 3.Sadii 4.Sadii oli |  |
| 207 | Daa’ima kee kan dhuma irratti deesse kana yeroo deesse rakkoon da’umsaan wal qabate simudatee turee? | 1. Eyyee 2. lakki |  |
| 208 | Yoo deebiin kee gaaffii 207 eyyee ta’e, rakkoolee kamfaatu si mudate? | 1. qaama hormaataan dhiiqni dhangala’uu.  2. bowwuu mataa cimaa.  3. ulfattinni qaamaa saffisaan dabaluu  4. Da’umsa mana yaalaatti taasisuu.  5. Miixuu/ciniinsuu sa’a dheeraa  6. Obbaattii/ofkaltiin ba’uu dhabuu.  7. kan biraa ibsi---------------------- |  |
| 209 | Da’umsa kee kan xumuraa kana irratti hordoffii kammiinuu taasifteettaa? | 1. eeyyee 2. lakki |  |
| 210 | Sababni ati gara mana yaalaa deemteef hordoffii da’umsa duraaf ta’e, si’a meeqa deemte? | Lakkoofsaan______________ |  |
| 211 | Hordoffii da’umsa duraa eessatti taasifte? | 1. hoospitaala  2. buufata fayyaa  3.kilinika dhunfaa  4. Kilinikaa mitmotumma  5. kan biraa, ibsi________________ |  |
| 212 | Yeroo Hordoffii da’umsa duraa taasifte odeeffannoo iddoo da’umsaa filachuu fi balaa hamaa yeroo da’umsaa argatteettaa? | 1. Eyyee 2. Lakki |  |
| 213 | Yoo eeyyee jette odeeffannoo akkamii?  (deebii tokkoo ol kennuun ni danda’ama) | 1. qaama hormaataan dhiiqni dhangala’uu.  2. bowwuu mataa cimaa.  3. ulfattinni qaamaa saffisaan dabaluu  4. Da’umsa mana yaalaatti taasisuu.  5. Miixuu/ciniinsuu sa’a dheeraa  6. Obbaattii/ofkaltiin ba’uu dhabuu.  7. kan biraa ibsi. |  |
| 214 | Ulffii kee inni dhuma kanaa karoora kee turee? | 1. Eyyee  2. Lakki |  |
| 215 | Daa’ima kee dhumaa eessatti da’uu akka qabdu kan murteessee eenyu ture? | 1. Anuma mataa koo  2.Abbaawarraa koo  3. Lamaan keenya  4. Ekisteenshinii fayyaa  5.kan biraa, adda baasi------------------------ |  |
| 216 | Mala kamiin deessee | 1.Gadameessaan  2.Garaa baqaqsaan  3.Mees haadhaan gargaara manii na deessisanii  4.Qaama saalaa kutanii/baqaqsanii hodhuun |  |
| 217 | Ogeessi sideessise/te dhiira moo dhalaa dha? | 1. Dhiira 2. Dhalaa |  |
| 218 | Dhabbata fayyaa keessaatti dahuuf maliif filtee? | 1.Tajaajila foyya’aa argachuuf  2. Bu’aa foyya’aa ofii kooti fi mucaa kottif argachhuf.  3.Muxxannoo badaa/gadhee/ manatti dhaluu yeroo darbee  4.Dhabbata fayyatti akkan dhaluu natti himameeti  5. Dhabbanni fayyaa mana kotti dhihoo jira.  6. Kan biro yoo jiratee ibsi-------------------- |  |
| 219 | Dhabbata fayyatti yoo deessee, dhabbataa fayyaa kamiitti deessee? | 1.Bufata fayyaa  2.Hospitaala |  |
| 220 | Sa’atii cinsuun sirra ture | 1. sa’a 6 gadii  2. sa’a 6- 12:00  3. sa’a 12- 24:00  4. sa’a 24 ol |  |
| 221 | Xumura/rawwii ulfaa isa dhihoo | 1. Lubbuu qaba.  2.kan du’ee/lubbu kan hin qabne |  |

Kutaa 3 ffaa: Gaaffilee ilaalchaa qulqullina tajaajila da’umsaa dhaabbata fayyaa irratti dhiyaatan.

| Lakk | **Dhaabbata fayyaa** | | | | | |
| --- | --- | --- | --- | --- | --- | --- |
|  | Gaaffilee | Sirriitti ittin walii gala | Ittin waliigala | yaada hin qabu | itti walii hin galu | sirriitti itti walii hin galu |
| 301 | Akka yaada keetti, baayyinni hojjettoota dhaabbata fayyaa keessa hojjetanii ga’aa dha. | 5. | 4. | 3. | 2. | 1. |
| 302 | Akka yaada keetti, baayyinni hojjettoota dhaabbata fayyaa ati itti fayyadamte keessa hojjetanii rakkoolee haadholii yaaluuf mijatoo dha. | 5. | 4. | 3. | 2. | 1. |
| 303 | Akka yaada keetti, bakki turanii dabaree eegan, kutaan sakatta’insaa ykn qorannoo yaalaa fi kutaaleen kan biroon dhaabbata fayyaa rakkoo haadholii furuuf ga’aadha. | 5. | 4. | 3. | 2. | 1. |
| 304 | Akka yaada keetti, dhiyeessi bishaan dhugaatii qulqulluu, bakki harka itti dhiqatanii fi manneen boolii ykn Fincaanii dhaabbata fayyaa keessatti argaman haadholiidhaaf ga’aadha. | 5. | 4. | 3. | 2. | 1. |
| 305 | Akka yaada keetti, walumaagallli naannoo dhaabbata fayyaa kanaa baayyeee qulqulluudha. | 5. | 4. | 3. | 2. | 1. |
| 306 | Akka yaada keetti, meeshaleen dhaabbata fayyaa kanaa keesatti argaman rakkoo haadholii sirriitti adda baasuuf kan sadarkaan isaanii eegamedha. | 5. | 4. | 3. | 2. | 1. |
| 307 | Fageenyi mana kee fi dhaabbata fayyaa kana gidduu jiru baayyee fagoodha. | 5. | 4. | 3. | 2. | 1. |
| 308 | Akka yaada keetti, hojjettoonni dhaabbata fayyaa kanaa haadholii yeroo ulfaafi da’umsa booda sirriitti qoratu/sakatta’u. | 5. | 4. | 3. | 2. | 1. |
| 309 | Dhaabbanni fayyaa kanaa sakatta’insa qaama hormaataafi tajaajila da’umsaa bakka mijataa fi namni nama arguu hin dandeenyetti kenna. | 5. | 4. | 3. | 2. | 1. |
| 310 | Akka yaada keetti, hojjettoonni dhaabbata fayyaa kanaa qorichoota barbaachisan ajaju/barreessu. | 5. | 4. | 3. | 2. | 1. |
| 311 | Akka yaada keetti, qorichoonni dhaabbata fayyaa kanaan kennaman gaariidha/baroodha. | 5. | 4. | 3. | 2. | 1. |
| 312 | Akka yaada keetti, haadholiin qoricha haaluma salphaan dhaabbata fayyaa kanarraa argachuu danda’u. | 5. | 4. | 3. | 2. | 1. |
| 313 | Sababa duraa duuba gochaalee hin barbaachisne yeroo da’umsaatiin miirrii nuffisiisaa fiqaaneffacuu natti dhagahameera. | 5. | 4. | 3. | 2. | 1. |
| 314 | Akka yaada keetti, odeeffannoo/hubannoo mallattoo balaa cimaa yeroo da’umsaa fi da’umsa boodaa hojjettoonni fayyaa kennan ga’aadha. | 5. | 4. | 3. | 2. | 1. |
| 315 | Akka yaada keetti, hojjetoonni fayyaaa dhaabbata fayyaa kana keessa hojjetan,rakkoo fayyadamtootaa sirriitti adda baasuu nii danda’u. | 5. | 4. | 3. | 2. | 1. |
| 316 | Akka yaada keetti, hojjettoonni dhaabbata fayyaa kana keessa hojjetan haadholiififtoomina baayyee dansaa/gaarii qabu. | 5. | 4. | 3. | 2. | 1. |
| 317 | Akka yaada keetti, hojjettoonni dhaabbata fayyaa kana keessa hojjetan haadholiif baayyee dhimmamoodha. | 5. | 4. | 3. | 2. | 1. |
| 318 | Akka yaada keetti, hojjettoonni dhaabbata fayyaa kanaa haadholiif sirriitti ni kabaju. | 5. | 4. | 3. | 2. | 1. |
| 319 | Akka yaada keetti, yeroon hojjettoonni fayyaa haadholiif qoodan ga’aadha. | 5. | 4. | 3. | 2. | 1. |
| 320 | Akka yaada keetti, hojjetoonni fayyaaa dhaabbata fayyaa kana keessa hojjetan sirriitti amanamoodha. | 5. | 4. | 3. | 2. | 1. |

Gaaffilee sassaabbii ragaa qabeenyaa Manneenii

| Kutaa 1- Ragaa qabeenyaa. Armaan gaditti meshaalee mana keessan keessatti argamanu isinan gaafadha | | | |
| --- | --- | --- | --- |
| 401 Mana kana keessa meshaalee armaan gadii kessaa kamtu jiraa ? yoo jiraatee (1) tti yoo hin jirre (0) tti mari | | Eyyee(1) | Hinjiru (0) |
|  | Raadiyo/CD/”tape recordarii’ hojjetu | 1 | 0 |
|  | Televisi’onii hojjetu | 1 | 0 |
|  | Stoovii/gaazii/ ibsaa elektrikaa | 1 | 0 |
|  | motorsaaykilii | 1 | 0 |
|  | Gaarii fardaa | 1 | 0 |
|  | Sa’aatii girgiddaa | 1 | 0 |
|  | Mobaayilii | 1 | 0 |
|  | ‘Soofaa’ | 1 | 0 |
|  | Firaashii spoonjii | 1 | 0 |
|  | Firaashii cidii | 1 | 0 |
|  | Genereetarii | 1 | 0 |
|  | Tiraaktara Qonnaa | 1 | 0 |
| 402 | Manni kun bineelda manaa armaan gadii qabaa? | 1.eyyee 0.hinqabu | Meeqa? |
|  | sangaa | 1.eyyee 0.hinqabu |  |
|  | Sa’a | 1.eyyee 0.hinqabu |  |
|  | Farad/gaangee | 1.eyyee 0.hinqabu |  |
|  | Hoolaa/ reetii | 1.eyyee 0.hinqabu |  |
|  | Reetii | 1.eyyee 0.hinqabu |  |
|  | Harree | 1.eyyee 0.hinqabu |  |

| 403 | Maatiin keessan bishaan dhugaatii eessaa argataa? | 1. bishaan boollaa itti ijaarame  2. bishaan boollaa itti hin ijaaramin  3. burqituu itti ijaarame  4.burqituu itti hin ijaaramin  5.Bishaan Biirii  6. bishaan bollaa paampii kan uummataa  6.Bishaan lagaa yaa’u  7.Ujummoo/sarara bishaanii dallaa keessaa  8. Ujummoo/sarara bishaanii dallaan alaa  Kan biro,ibsi ___________ |  |
| --- | --- | --- | --- |
| 404 | Maatiin keessan mana fincaanii akkamiitti fayyadama? | Boolla qotamaatti  Boolla fincaanii dahannaa qabu  Boolla fincaanii dahannaa hin qabne  Mana fincaanii sadarkaa isaa eeggate  Dirree,ykn bakkee irratti  Kan biraa,ibsi____________ |  |
| 405 | Mana fincaanii kana maatiin kan biraa isinwaliin nifayyadama? | Eeyyee 0/ Lakki | Yoo lakkii ta’e gara gaaffii 407 tti darbi |
| 406 | Yoo deebiin 405 eeyyee ta’e Baayyinni Abbaa warraa isin waliin itti fayyadamanii meeqa? | Baayyina abbaa warraa_______________ |  |
| 407 | Hundeen lafa mana kanaa maalii?.Ilaalii /daawwadhu mirkaneessi. | Biyyoo/ lafa  Dikee /compostii  Muka  Simintoo  Kan biro ________________ |  |
| 408 | Ijoon (Uwwisi)) mana kanaa maali?  Ilaalii/daawwadhuu mirkaneessi. | Uwwisa hin qabu  Citaa ykn baala  Sibiila qorqorroo  Kan biraa___________________ |  |
| 409 | Duppon ykn Gidgiddaan mana kanaa maal irraa tolfame?  Ilaalii/daawwadhuu mirkaneessi. | Natural walls  Keenyan hin qabu  Mukaafi biyoo ykn Dhoqqee  Suphee ykn shakilaa duudaa hin ta’in  Bilookeetii ykn Shakilaa duudaa  Muka ykn xawulaa hin dulloomne (yeroo birraaf kan fayyadu)  Kan biraa___________________ |  |
| 410 | Manni keessan kun kutaa meeqa qaba? | Baayyina kutaa________ |  |
| 411 | Maatii keessan lafaqotisaa hagam qaba ? | Safartuu naannoo(hektaara) ---------------  2. Hin beeku |  |
| 412 | Maatii keessan keessaa namni accountii baankii ykn baankii qusanoo fayyadamu jiraa? | Eeyye  Hinjiru |  |
